# Exploring the Dimensions of Smartphone Distraction: Development, Validation, Measurement Invariance, and Latent Mean Differences of the Smartphone Distraction Scale (SDS)

**DOI:** 10.3389/fpsyt.2021.642634

**Published:** 2021-03-08

**Authors:** Melina A. Throuvala, Halley M. Pontes, Ioannis Tsaousis, Mark D. Griffiths, Mike Rennoldson, Daria J. Kuss

**Affiliations:** ^1^International Gaming Research Unit, Psychology Department, Nottingham Trent University, Nottingham, United Kingdom; ^2^Department of Organizational Psychology, Birkbeck, University of London, London, United Kingdom; ^3^Department of Psychology, University of Crete, Rethymnon, Greece; ^4^Psychology Department, Nottingham Trent University, Nottingham, United Kingdom

**Keywords:** smartphone use, distraction, attention, social media use, smartphone distraction scale

## Abstract

**Background:** Distraction is a functional emotion regulation strategy utilized to relieve emotional distress. Within the attention economy perspective, distraction is increasingly associated with digital technology use, performance impairments and interference with higher-order cognitive processes. Research on smartphone distraction and its association with problematic smartphone use is still scarce and there is no available psychometric assessment tool to assess this cognitive and emotive process parsimoniously.

**Method:** The present study reports the development and evaluation of the psychometric properties of the Smartphone Distraction Scale (SDS) through exploratory and confirmatory factor analysis, construct validity, gender invariance, and latent mean differences. The study was conducted in a sample of British university students (*N* = 1,001; *M* = 21.10 years, *SD* = 2.77).

**Results:** The 16-item SDS was best conceptualized in a four-factor model solution comprising *attention impulsiveness, online vigilance, emotion regulation*, and *multitasking*. Construct validity was established using relevant psychosocial and mental health measures, with SDS scores being moderately associated with deficient self-regulation and problematic social media use. Gender measurement invariance was achieved at the configural, metric, and scalar levels, and latent mean differences indicated that females had significantly higher means than males across all four SDS latent factors.

**Discussion:** The SDS presents with several strengths, including its theoretical grounding, relatively short length, and sound psychometric properties. The SDS enables the assessment of distraction, which appears to be one of the pathways to problematic smartphone use facilitating overuse and overreliance on smartphones for emotion regulation processes. The assessment of distraction in relation to problematic use in vulnerable populations may facilitate interventions that could encourage metacognition and benefit these groups by allowing sustained productivity in an increasingly disrupted work and social environment.

## Introduction

Attention is a scarce finite resource implicated in a variety of cognitive processes determining individual action and volition (1) that can be deployed externally (e.g., focus on the shape of a certain stimulus) or internally (e.g., focus on neutral or positive thoughts) (2). In the digital age, and particularly in the current pandemic era, which has shifted education and employment to remote learning and working, respectively, attentional resources are consistently challenged for engagement (3, 4). Concerns have been raised that the increased pressures for digitally juggling remote working with social, recreational, and information demands may be contributing to difficulties maintaining a healthy work-life balance (5) and the onset of mental health difficulties such as occupational burnout (6, 7). Additionally, online social spaces are influencing users with persuasive design (i.e., rolling feeds), prompting high cue reactivity and prolonged use of and overreliance on digital devices (8–11). Multitasking, multiple device use, and frequent attentional shifts are salient behaviors potentially leading to digital information overload (12–14).

Smartphones are ubiquitous digital devices that offer multiple communication affordances to half of the world's population (15), and may interfere with how attentional resources are allocated, constituting an emerging area of research (16–19). Increasing evidence suggests that smartphone use triggers frequent interruptions and breaks from main tasks, further interfering with cognitive processes and ability (20–24), cognitive functioning (25–28), and associated with distraction and compromised performance (26–28) resulting in sub-optimal learning among young people (29, 30). Disruption from smartphone use is even more prominent within classroom environments (31–33), hindering academic achievement due to interference with primary tasks (12, 34) and in less engaging academic contexts, prompting lower motivational levels and comprehension (12, 35, 36), task performance (37), and chronic media multitasking (12). Smartphone interruptive notifications are frequent external triggers (38) which disrupt daily activities and have even been associated with mood disorders mediated by boredom proneness (39).

Given the numerous advantages of smartphones which provide constant internet accessibility, distraction has become frequent and endemic among smartphone users, potentially reinforcing more habitual or compulsive smartphone use (40). Distraction has been traditionally defined as an emotion regulation coping strategy implicated in shifting focus to a non-threatening situation or thought to reduce emotional distress and negative affect (41–45). Smartphone distraction (SD) may be caused by external triggers, such as notifications, intrusive thoughts, or cognitive salience of smartphone-related content to avoid or regulate emotions (26, 46–48). Fear of missing out (FOMO: missing out on positive recreational experiences of others) appears to be a main driver for several forms of problematic technology use (49), including smartphone use (50) currently exacerbated by the impact of the pandemic and social isolation (51) and driving attentional bias and distraction from online content to fulfill control needs (52).

**Figure 1:**
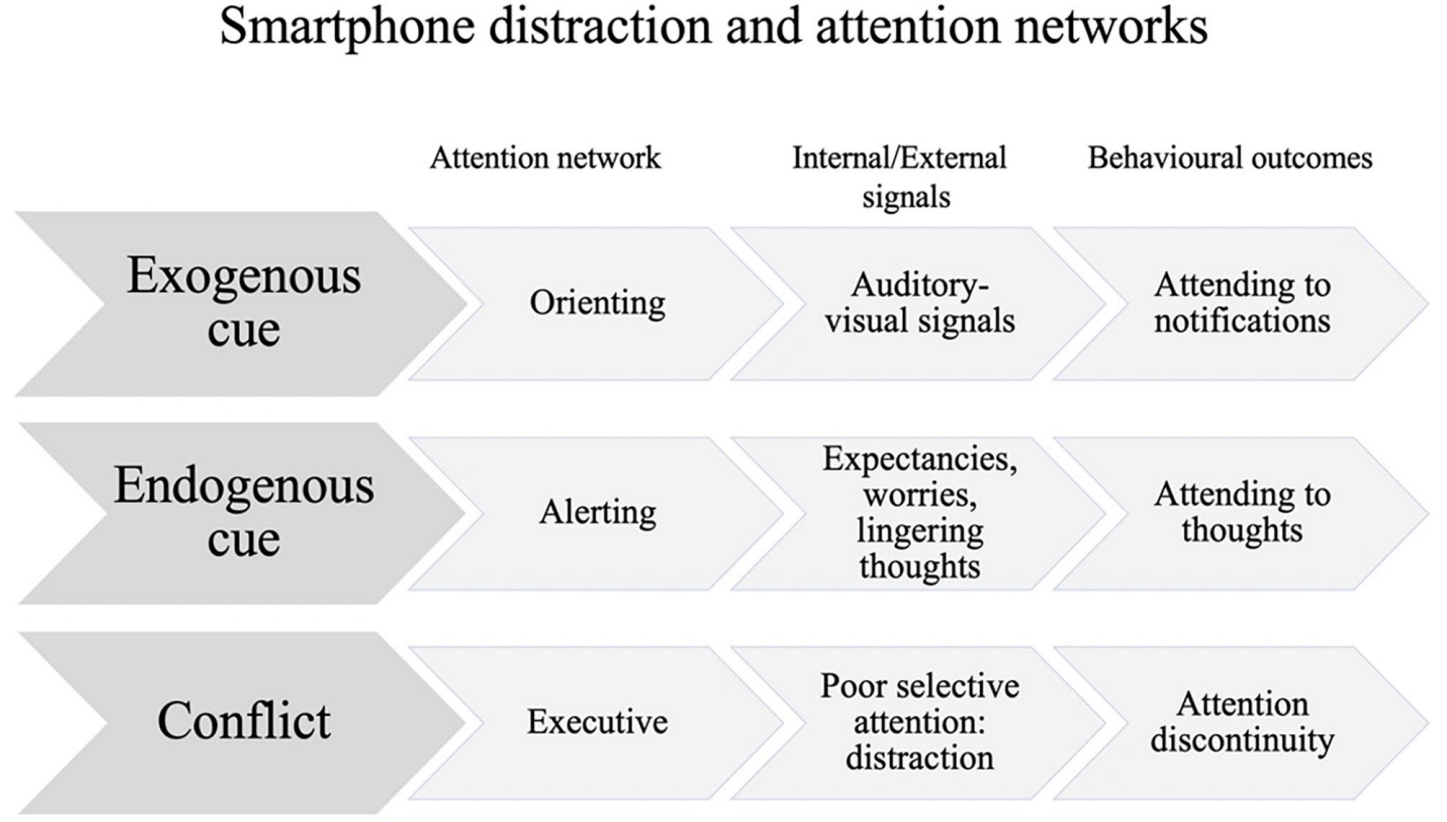
Posner's attention model (53) adapted by Wu and Cheng (54) and further adapted for smartphone distraction.

One of the most prominent models of attention and its orientation has been proposed by Posner (53), viewing the attentional system as having the possibilities to shift, orient, and disengage as a biased response. Based on Posner's attention networks model (53), as adapted by Wu and Cheng (54) for educational contexts (see Figure 1), SD is conceptualized within the present study as the result of a reaction to exogenous (orienting system) or endogenous cues (alerting system) or as the result of a conflict amongst these two networks that are competing for attentional resources. For the occurrence of distraction, the exogenous cues (orienting system) are triggered by auditory/visual signals, which can take the form of smartphone notifications in smartphone use. The endogenous cues (alerting system) are the bottom-up signals in the form of expectancies, worries, and lingering thoughts leading to distraction or daydreaming. The executive system is implicated when conflict arises between the exogenous and endogenous cues, leading to attention discontinuity and therefore poor attention deployment, prompting inhibitory or executive control difficulties (55). Distraction appears therefore to be the result of disruptions or interruptions in one of the three attention networks mediated by smartphone use (29, 39, 54).

Distraction may be psychologically explained by the control model of engagement (52), a theoretical model integrating elements from distraction conflict theory (56), theory of social facilitation (57), and perceptual control theory (58), supporting that online engagement partially occurs to control online content, relationships and presentation online, causing attentional bias toward online stimuli and distraction from daily activities. Distraction may be facilitated by the presence of others online (56, 59), prompting interaction and leading to heightened engagement or shallow processing when involved in parallel cognitively demanding tasks. Beyond perceptual conflicts (12, 34) associated with lowered levels in well-being and productivity or lowered academic achievement amongst young people (31, 60–63) due to excessive social media and smartphone use (64–66), these constant disruptions may be associated with hyperactivity levels (67), negative affect, sensitivity to evaluation, poor emotion regulation, and problematic smartphone use (68–74). Attempting to achieve relief from negative emotions elicited smartphone use is reinforced (75, 76), leading to poor metacognition (77). However, despite accruing evidence for emotional and behavioral consequences of problematic smartphone use, the processes leading to addictive use (78) remain conceptually unclear and methodologically questionable partially due to the constantly evolving nature of products and services (17, 79–82) alongside the wide range of contents (social, information) smartphones provide access to.

Research on distraction and its association with problematic smartphone use is still scarce and there are no available psychometric assessment tools within the smartphone and social media literature to assess this cognitive and emotive process parsimoniously. Subscales within attention scales, executive function scales, and problematic internet use scales partially assess the role of distraction as a cognitive mechanism occurring in the digital environment (83–85). However, many of the existing psychometric scales are limited to a few items only, and therefore are neither comprehensive nor representative of the complexity involved in smartphone use experience, frequent attentional loss, and the associated processes experienced by smartphone users (i.e., urge to check, cue reactivity). Given that frequent attentional loss has been reported to affect executive function areas, critical for paying attention, decision-making, planning, organization, higher-order thinking, and regulating emotions (86, 87), it is important to assess distraction within the smartphone context with accuracy. Thus, the psychological function of distraction in the online environment should be further scrutinized since distraction is not a unitary process, but rather a multidimensional construct associated with both adaptive and maladaptive functions, rendering the development of such psychometric test timely due to the need to further understand this phenomenon and its relationship to problematic smartphone use as a psychological experience.

## Smartphone Distraction and Relevant Psychological Constructs

Smartphone distraction among young people primarily occurs due to social media content. Smartphone use and social media use are inextricably interwoven for young people due to the prominent social element in smartphone use (88) leading to distraction and academic work conflict (89). More specifically, the rationale for the development of this scale was based on the premise that distractive smartphone use appears to be driven primarily by the cognitive preoccupation with social media content in order to attend to needs for validation and control (of content, self-presentation, and relationships). This preoccupation and urge to check (90) or interact, in turn, prompts emotional reactivity and behavioral activation in the form of distraction (40, 91), amplified by FOMO and the need to control self-presentation and others' perceptions or seek reassurance (92). This process could also be experienced from non-social use (73, 93) because smartphones are multi-purpose devices and recent studies suggest that process use (e.g., watching videos, browsing online) is widespread as much as social use (73) and with stronger associations with problematic smartphone use (92, 94). In the present study, it is contended (and supported by empirical studies) that social media content is largely responsible for the attentional drift associated with frequent and prolonged smartphone engagement among young adults (95, 96). A smartphone is therefore viewed as the medium providing access to the desired content reflecting the attachment formed to the device among young adults (97–103) and intensified by experiences of nomophobia (NOMO; the fear of being without a smartphone) (104–107) and FOMO (29, 108, 109). However, the assessment of the relative role of process smartphone use and its relationship to smartphone distraction requires further exploration. Given the increasing mobile connectivity, providing access to social media via smartphones (110), and the frequent engagement with social content by emergent adults (111, 112), the use of social media measures (metacognitions and problematic social media use) were deemed appropriate to support the validity of the new measure.

### Metacognitions

*Metacognitions* refer to higher order cognitive states and coping mechanisms to regulate those cognitions (113). These refer to *positive* cognitive-affective regulation (i.e., “*Smartphones distract me from worries*”) and negative metacognitions (i.e., “*I am unable to control my distraction*”) which denote the inability to control a cognition or a behavior and may amplify maladaptive engagement (113). A bi-directional association between distraction and metacognition has been established for auditory distractions, suggesting interference of distraction in metacognition and vice versa (114). Within the context of gambling, negative metacognitions have been associated with attention focusing and attention shifting and have been suggested as partially influencing the control of attention (115). As recently evidenced in the literature, both positive and negative metacognitions for emotion regulation, social benefits, and inability to control behavior have been found to predict problematic smartphone use (116) and have been associated with problematic social media use (113, 117) and problematic internet use (118). Metacognitive processes were chosen for construct validity due to evidence implicating such processes in problematic smartphone use and because they may also serve as a potential pathway to controlling problematic social media use (113) through positive beliefs about cognitively controlling attention (115).

### Problematic Social Media Use

*Problematic social media use*, reflects a prolonged pathological engagement with social media content (119), which may be mediated by distraction and constant checking (11, 40, 83, 120). The current literature suggests that frequent smartphone checking behaviors (91, 121) have been associated with distraction (46, 122, 123) and habitual use (94, 121) fueled by FOMO, neurotic tendencies (124) and online vigilance (i.e., preoccupation with salient online content) (91). Therefore, experiences of FOMO and NOMO appear to be associated with distraction and may be driving checking behaviors (125), reflecting the cognitive preoccupation and interpersonal attachment via digital devices (10, 68, 126–131). Positive metacognitions also appear to mediate the relationship between FOMO and problematic social media use (132). Therefore, within smartphone use, distraction reflects a salient cognitive and emotive coping strategy, mediating or facilitating other potentially problematic processes in smartphone use (e.g., checking behaviors) or facilitating higher engagement for emotion regulation (40). Therefore, investigating the role of SD alongside its role in distress and problematic smartphone use (133) via problematic social media use (95, 134–136) and its differentiation from similar constructs (i.e., mind-wandering, interruptions) (137, 138), is timely because it is the context (smartphone use) and the function which accounts for the renewed scientific interest in the construct. The present authors utilize the term “problematic social media use” (similarly to “problematic smartphone use”) instead of “social media addiction” given that the latter is not currently a formally accepted diagnostic construct (139) and respective screening measures reflect problematic engagement. “Social media addiction” as a term will only be used in the present manuscript where referenced in other studies. Social media addiction is a construct used by scholars to denote a state of addictive proclivity to social media when meeting criteria for addiction (140) with an evolving literature base regarding its nature and impact cross-culturally and longitudinally (141–144).

## Gender Differences In Smartphone Use

Prior studies have confirmed gender differences in emotional distraction and reactivity (145). Within smartphone use, emergent evidence has also demonstrated gender-based differences with empirical studies to date presenting with mixed results concerning gender differences in smartphone use (124, 146–154). Gender has also been arguably identified as a potential risk factor for the development of problematic smartphone use with more females reporting higher problematic smartphone use than males but also gender differences in social media and other smartphone-related behaviors (155–157). However, given the novelty of the construct, gender differences have not been examined in relation to smartphone distraction. Therefore, a multiple group confirmatory factor analysis was undertaken to assess measurement invariance (configural, metric, and scalar) of the Smartphone Distraction Scale (SDS) across gender, and investigate gender-related latent mean differences across all the identified latent factors. Based on the analysis of the current literature, higher scores for smartphone distraction were expected for females than for males.

The present study therefore aimed to develop and empirically validate a psychometric scale to assess smartphone distraction (SD), the SDS. This was developed to identify its latent dimensions while accounting for the smartphone context, the extant empirical evidence, and the theoretically-relevant frameworks suggested (52, 58). More specifically, the present study aimed to fulfill the following primary objectives: (i) examine the factorial validity and reliability of the SDS using exploratory and confirmatory factor analysis, and (ii) investigate the convergent and divergent validity by examining the relationship between the SDS and problematic social media use, metacognition, mindful attention, stress and smartphone-related psychological constructs. To achieve the aforementioned objectives, it was hypothesized that: (i) the SDS would show robust psychometric properties; and (ii) those with higher levels of distraction would present higher scores of problematic social media use, stress, and other relevant psychological constructs (i.e., self-regulation). It is envisaged by the present authors that the development and psychometric validation of a scale for SD will contribute to its assessment in academic institutions and work-related environments, generating further multidisciplinary scientific knowledge about this disruptive construct and its relationship with mental health correlates in smartphone use.

## Methods

### Scale Development

The psychological dimensions of SD informed the item pool reflecting the following dimensions: (i) behaviors related to attention impulsiveness due to notifications or even the mere presence of a smartphone, (ii) preoccupation with online content, frequent checking, FOMO and NOMO, (iii) use of a smartphone to regulate distress, and (iv) multitasking and interference in daily activities and face-to-face interactions. This psychometric test was developed primarily for use with young adults (i.e., university students) who are the most frequent users of smartphones and therefore the most likely to experience academic disruption caused by smartphones with heightened distraction levels in University settings (34, 61, 158, 159) and subsequent attentional losses due to smartphone use (31, 34, 160).

An initial pool of 36 items was generated with attention to double-barreled items, leading questions, reverse-scored items, and clear short item presentation (161). Items were reviewed in terms of their conceptual relevance, coherence, linguistic clarity, and adequacy, by: (i) a panel of expert psychologists from the fields of cyberpsychology, behavioral addictions, clinical psychology, and psychometrics, respectively, and (ii) a pilot-testing among 35 university students to assess face validity, comprehension, and relevance of the items. A final pool of 33 items (in Appendix 1) formed the scale with each item rated on a 5-point Likert scale ranging from 1 (*almost never*) to 5 (*almost always*), which corresponded to four hypothesized factors. Following this initial step, the scale's dimensionality, validity, reliability and invariance was psychometrically assessed, following a stepwise approach as suggested by scholars (162, 163).

### Participants and Procedure

An initial sample of 1,129 English-speaking university students from the United Kingdom (UK) were recruited online using snowball sampling. After data cleaning (see the “Statistical analyses” subsection), the sample was randomly split into two subsamples; the first sub-sample (Sample 1, *n* = 501) was used in Exploratory Factor Analysis (EFA) and the second one (Sample 2, *n* = 500) in Confirmatory Factor Analysis (CFA) to assess for population cross-validity (164, 165). Participant recruitment took place through university lectures in exchange for university credit as well as on social media with a potential financial compensation in the form of a prize draw of *Amazon* vouchers through a pool of eligible participants. The online survey was developed and administered via the survey platform *Qualtrics* (Provo, UT, USA) and included an information sheet, a consent form, and self-report questions to assess eligibility. Ethical approval for the present study was granted study by the University's Ethics Committee (No. 2018/226), and only participants who met the following inclusion criteria were able to complete the survey: (i) owning and using a smartphone with internet connection regularly for at least a year, (ii) using social media platforms on a daily basis, and (iii) being at least 18 years old. The survey took ~25 min to complete.

### Measures

*Socio-Demographics and Media Use Habits*. Socio-demographic and usage data were collected (gender, age, educational level, and relationship status) alongside data asking participants to indicate smartphone and social media use (average number of hours per day) on a multiple choice or open response format. Individuals also completed additional psychometric tests in order to assess the predictive ability of the new scale being developed (criterion-related validity).

*The Attentional Control Scale (ACS)* (166) is a 20-item self-report scale which assesses differences in the control of the orientation of attention as defined by three factors: attention focusing, attention shifting, and flexible control of thought (166, 167). Sample items in the scale include “*It is easy for me to read or write while I'm also talking on the phone*,” and “*I can become interested in a new topic very quickly when I need to*.” Items are rated on a 4-point Likert scale ranging from 1 (*almost never*) to 4 (*always*) with higher scores indicating greater difficulty to focus attention. Focusing attention has been associated with high anxiety and shifting attention with depression (166, 168). The ACS demonstrated adequate psychometric qualities in the present study (Cronbach's α = 0.80).

*The Mindful Attention Awareness Scale (MAAS)* (169) is a 15-item assessment scale that assesses the dispositional mindfulness of being open and receptive in what is occurring in the present. The construct has been psychometrically and experimentally validated on various demographics and has been associated with various well-being constructs (169, 170). Item statements assess mindfulness within everyday situations reflecting cognitive, emotional, and behavioral aspects of the construct. Items are rated on a 6-point Likert scale from 1 (*almost always*) to 6 (*almost never*) with higher averaged scores indicating higher levels of dispositional mindfulness. Sample items include “*I do jobs or tasks automatically, without being aware of what I'm doing*” and “*I find myself doing things without paying attention*.” The construct has demonstrated a high degree of internal consistency in the present study (Cronbach's α = 0.90).

*The Perceived Stress Scale (PSS)* (171) is a widely used 10-item scale assessing the degree of appraisal of life situations as unpredictable and beyond control causing additional burden to an individual. The construct has been associated with more severe negative affective states and the onset of diseases (172). All items are rated on a 5-point Likert scale from 0 (*never*) to 4 (*very often*) with sample items such as “*In the last month, how often have you felt nervous and stressed?*” and “*In the last month, how often have you been able to control irritations in your life?*” Higher scores indicate greater levels of perceived stress. The scale possesses good psychometric properties (173) and had adequate internal consistency in the present study (Cronbach's α = 0.68).

*The Barratt Impulsiveness Scale-Alternative Version (BIS-8)* (174) is an abbreviated version of the 11-item BIS scale (174) containing eight items assessing individuals' predisposition to fast and unplanned reactions with lack of control, and it is a construct associated with poor self-regulation and maladaptive behaviors (175). In previous studies the BIS-8 has presented with adequate levels of construct and concurrent validity among young populations (176, 177). Items are rated on a 4-point Likert scale ranging from 1 (*do not agree*) to 4 (*agree very much*) and higher mean scores indicate a higher degree of impulsiveness. Sample items include: “*I say things without thinking*” and “*I plan tasks carefully*.” In the present study, the BIS-8 had adequate levels of reliability (Cronbach's α = 0.77).

*The Deficient Self-Regulation Measure (DSR)* (178) is a 7-item scale assessing poor self-regulation in video game playing adapted for smartphone use (40) and unregulated internet use (179). This measure has been shown to exhibit sound psychometric properties (178), with sample items in the scale adapted for smartphone use including “*I get strong urges to use social media*” and “*I feel my social media use is out of control*.” Items are rated on a 7-point Likert scale ranging from 1 (*almost never*) to 7 (*almost always*), with grater scores suggesting higher levels of deficient self-regulation toward smartphone use. In the present study, the scale had adequate levels of reliability (Cronbach's α = 0.89).

*The Bergen Social Media Addiction Scale (BSMAS)* (180–183) is a 6-item scale assessing the risk of problematic and addictive social media use severity based on the framework of the components model of addiction (salience, mood modification, tolerance, withdrawal, conflict, and relapse) (140). Items are rated on a 5-point Likert scale ranging from 1 (*very rarely*) to 5 (*very often*), producing a composite score ranging from 6 to 30, with higher scores indicating greater risk of social media addiction severity. A cut-off score ≥19 indicates problematic social media use (184). Sample items from the BSMAS is “*How often during the last year have you … used social media in order to forget about personal problems?*” and “*How often during the last year have you … become restless or troubled if you have been prohibited from using social media?*” The BSMAS has demonstrated sound psychometric properties (180–183, 185). In the present study, the BSMAS had excellent levels of internal consistency (Cronbach's α = 0.84).

*The Metacognitions about Gaming Questionnaire (MGQ)* (186) was adapted for social media use for the present study. The 12 items are rated on a 4-point Likert scale ranging from 1 (*do not agree*) to 4 (*agree very much*). The MGQ includes two latent factors: positive metacognitions and negative metacognitions about social media use. Negative metacognitions refer to the difficulty in controlling social media use, content-related thoughts, and positive metacognitions to adaptive reflective beliefs related to cognitive and emotional responses to social media use. Sample items include “*Thoughts about social media interfere with my functioning*” and “*Social media stops me from worrying*.” Higher scores represent greater levels of metacognitions about social media use. The scale has demonstrated adequate psychometric properties in previous research (186). Internal consistency in the present study was excellent: for the positive metacognition subscale (Cronbach's α = 0.90) and for the negative metacognition subscale (Cronbach's α = 0.89).

*The Generalized Self-Efficacy Scale (GSE)* (187) is a widely used 10-item scale assessing perceived self-efficacy and is associated with both positive (i.e., optimism, work satisfaction) and negative outcomes (i.e., depression, stress, and anxiety). Sample items include: “*If I am in trouble, I can usually think of a solution*” and “*I can always manage to solve difficult problems if I try hard enough*.” All items are rated on a 4-point Likert scale ranging from 1 (*not at all true*) to 4 (*exactly true*). The GSE has demonstrated satisfactory internal consistency and validity in previous research (188, 189), and also high levels of internal consistency in the present study (Cronbach's α = 0.86).

### Statistical Analyses

The two subsamples were tested for equivalence with the use of independent samples *t*-tests and chi-square tests for socio-demographic variables. The constructs assessed indicating independence and Cohen's *d* designated trivial effect sizes. Statistically significant differences were found for age, gender, education, social media use, and problematic social media use (social media addiction). However, given the high sample size utilized in both subsamples, statistical significance may be inflated (190). Data cleaning involved identifying missing values above the 10% threshold for incomplete data, which resulted in 117 cases being excluded with listwise deletion based on literature suggesting that retaining data with missing data above this threshold may render biased results (191). To assess similar and repetitive patterns of responses (i.e., acquiescence bias) across the scales, Little's Missing Completely at Random (MCAR) test determined that data were missing completely at random (*p* = 0.617) in the remaining dataset. Multiple imputation was used to handle missing data. Univariate normality of all 33 items of the SDS was assessed by examining skewness and kurtosis values for each item. Three data points on the SDS had absolute values of skewness >3.0 and kurtosis > 8.0 (192), which were further removed from the dataset. Tolerance and Variance Inflation Factor (VIF) values suggested that there were no multicollinearity issues in the data. Mahalanobis distances and critical values for each case were used to check for multivariate outliers, resulting in eight cases being excluded from the dataset. Therefore, the final sample size for all subsequent analyses included 1,001 participants. Finally, to examine whether the assumption of multivariate normality was met, the Mardia index of multivariate skewness and kurtosis was applied. The Mardia's skewness for this data set was 253.44 and the Mardia's kurtosis 1,271.86. Both values are above the acceptable thresholds (i.e., 10 for multivariate skewness and *p*(*p*+2) for multivariate kurtosis, which for our data was 288), indicating that the data were not multivariate normally distributed (193). All analyses were performed using Mplus v.8.3 (194).

#### Exploratory and Confirmatory Factor Analysis, Reliability, and Validity of the SDS

Statistical analyses involved: (i) estimation of descriptive statistics of the sample, (ii) an EFA to explore the underlying structure of the SDS, and (iii) CFA to ascertain the latent dimensions of the main construct, and to estimate the fit of the latent factors as defined by the EFA (195). This was decided because even though the items of the SDS being tested were defined *a priori* (based on the literature review of general distraction, the smartphone literature, and the expert comments), the lack of any relevant scale assessing this construct demanded an initial exploration of hypothesized theoretical factors, which would be further tested for their validity. In the EFA, Principal Axis Factoring extraction method was used with Promax (oblique) rotation due to the assumption that the factors are correlated, based on the underlying conceptual framework assumed (196). To measure sampling adequacy and suitability of the data for factor analysis, Bartlett's test of sphericity (BTS) and the Kaiser-Meyer-Olkin (KMO) measure were computed (197). A scree plot was also used to visually determine the number of factors to be retained (198) using the Kaiser criterion [retaining all factors with eigenvalues >1; (199) to obtain the most viable factor solution] (200, 201). To address criticisms of the Kaiser criterion technique (200, 202, 203) related to overestimation of the true number of factors (204), Horn's Parallel Analysis (205) was also performed since it is considered one of the most accurate factor retention methods and a better technique (206) based on the Monte Carlo simulation process, simulating random samples that parallel the observed data (207).

For the CFA, the following recommended fit indices with the conventionally accepted cut-off values were used to assess the fit: Root Mean Square Error of Approximation (RMSEA) [0.05;0.08], Standardized Root Mean Square Residual (SRMR) [0.05;0.08], Comparative Fit Index (CFI), Tucker-Lewis Fit Index (TLI), and Goodness of Fit Index GFI [0.90;0.95]. Maximum likelihood with robustness to non-normality and non-independence of observations (MLR; [194]) was used as the method of estimation for all models. Analysis of the reliability of the SDS was performed using two different indicators of internal consistency (McDonald's Omega and Cronbach's alpha). The validity of the scale was evaluated using several types of validity indicators such as criterion, convergent and discriminant validity (162, 163) by assessing the association between the SDS and measures of relevant psychological constructs (i.e., attentional control, and generalized self-efficacy).

### Gender Invariance and Latent Mean Differences

Gender invariance was performed to assess similarity or divergence in the interpretation of the construct across gender and identify any latent mean differences across the factors. The present study also tested alternative models of fit by testing for invariance across gender, which was deemed critical given the multidimensional nature of the construct, influenced by individual differences in smartphone use (148, 154, 208). The invariance testing process begins with a well-fitting baseline model and involves the testing of equality of sets of parameters through several ordered and progressively more restrictive steps in measurement invariance by testing equality (209, 210). To assess gender invariance, a multi-group CFA (MGCFA) was conducted with maximum likelihood estimations to assess model fit by comparing fit indices amongst the models (209). Invariance may be achieved if there is an adequate fit to the data across groups with only a negligible change in values for fit indices (e.g., ΔCFI and ΔRMSEA, or ΔSRMR) (211). Three models—configural invariance, metric or weak invariance, and scalar or strong invariance—were estimated.

Traditionally, gender differences have been investigated using *t*-tests or analysis of variance comparing composite scores. However, a superior analytical method to examine gender differences is the latent mean analysis, which considers comparisons across groups based on a construct's latent factors, which cannot be directly measurable (212). In a SEM framework, to estimate the difference between two group means at a latent level, one of the groups should be served as a reference group and its mean should be fixed to zero. In this case, the latent mean of the other group represents the difference between the latent means of the two groups. “Males” was chosen as a reference group (coded as 0). In practice, the difference between the two group means on each latent variable equals the mean of the non-reference group (females) on the latent construct. Thus, a significant mean of a compared group would indicate that this group has a different level of the latent construct relative to the reference group. It is important to note that (full or partial) scalar invariance is a prerequisite in order to test for latent mean differences (212, 213).

## Results

### Descriptive Statistics

The final sample of 1,001 English-speaking smartphone and social media users was predominantly female (69%, *n* = 690), 30% male (*n* = 300), and 1% other (*n* = 11) with an age range from 18 to 30 years (*M*_age_ = 21.10 years, *SD* = 2.77). A total of 730 participants (72.9%) were undergraduate students, 95 were graduate and post-graduate students (9.4%), 76 (7.6%) were employed and 28 (2.8%) participants were unemployed, whereas 72 (7.2%) were both students and employees. Sample 1 (*n* = 501) consisted of 88 (17.6%) males, 411 (82.2%) females, and two (0.2%) participants who declared as gender-free, whereas Sample 2 (*n* = 500) consisted of 212 (42.4%) males, 279 (55.8%) females, and nine (1.8%) participants who declared as gender-free. The two samples presented with the following composition in terms of ethnicity Sample 1 (*N* = 501), White, 369 (73.7%), Black, 44 (8.8%), Asian 30 (6%), and other 58 (11.6%). Sample 2 (*N* = 500), had a similar composition, White, 320 (64%), Black, 56 (11.2%), Asian 45 (9%), and other 79 (15.8%). More than half of the participants (*n* = 524, 52.3%) were in a relationship and reported different levels of daily smartphone usage: 305 (30.5%) from half an hour to 3 h (0.5–3 h), half of the participants (*n* = 503, 50.2%) reported 3–6 h of smartphone use (3–6 h), 158 (15.8%) participants (6–10 h), and 35 (3.5%) of participants reported (10h+) of smartphone use.

### Psychometric Properties of the Smartphone Distraction Scale

#### Exploratory Factor Analysis

An EFA was conducted on all SDS items in Sample 1 (*n* = 501) to examine the factorial structure and construct validity (195, 196) of the scale. Sample 2 (*n* = 500) was utilized to conduct the CFA for testing the findings from the EFA and to corroborate the factor structure emerging from the EFA (196). Results indicated that the proportion of variance in the variables explained by underlying factors was sufficient to indicate a strong relationship and conduct a factor analysis on the data (KMO = 0.854; BTS [χ^2^[120, 501] = 2.597,36, *p* < 0.001). Following conventions in EFA, items with factor loadings <0.40 were not retained (214). The communalities suggested that each item shared some common variance with other items and ranged from 0.20 (i.e., Item 21) to 0.62 (i.e., Item 30), meeting the thresholds to retain items and interpreted to be indicative of that factor (215).

The initial eight-factor solution was not retained as it rendered factors with fewer than three indicators and was an overestimation of the factors with no meaningful theoretical interpretation (196, 201). Parallel analysis also indicated a four-factor solution. Furthermore, the EFA analysis suggested a four-factor structure that was extracted after six iterations, explaining about 59.62% of the total variance of the construct (see Table 1). A four-factor solution was corroborated by this analysis (four factors emerged with an eigenvalue above 1), which was a manifestation of the multidimensionality of the construct.

**Table 1:** Summary of the results from the Exploratory Factor Analysis (EFA) on the SDS 33 items obtained from Sample 1 (*n* = 501).

**Items**	**Factor Loadings**				**Communalities**	
	**F1** **(ω = 0.78)** **(α = 0.84)**	**F2** **(ω = 0.74)** **(α = 0.80)**	**F3** **(ω = 0.83)** **(α = 0.74)**	**F4** **(ω = 0.63)** **(α = 0.75)**	**Initial**	**Extraction**
**Factor 1: Attention Impulsiveness (F1)**						
Dis2: I get distracted by my phone apps	0.796				0.488	0.532
Dis1: I get distracted by my phone notifications	0.735				0.509	0.605
Dis3: I get distracted by just having my phone next to me	0.720				0.485	0.560
Dis4: I get distracted by my phone even when my full attention is required on other tasks	0.622				0.531	0.575
**Factor 2: Emotion Regulation (F2)**						
Dis30: Using my phone distracts me from tasks that are tedious or difficult		0.782			0.497	0.620
Dis27: Using my phone distracts me from doing unpleasant things		0.688			0.374	0.433
Dis28: Using my phone distracts me from negative or unpleasant thoughts		0.637			0.347	0.395
Dis31: Using my phone distracts me when I'm under pressure		0.634			0.405	0.445
**Factor 3: Online Vigilance (F3)**						
Dis16: I get distracted with what I could post while doing other tasks			0.690		0.386	0.488
Dis7: I get anxious if I don't check messages immediately on my phone			0.643		0.369	0.416
Dis13: I think a lot about checking my phone when I can't access it			0.641		0.455	0.516
Dis17: I get distracted thinking how many likes and comments I will get while doing other tasks			0.553		0.311	0.342
**Factor 4: Multitasking (F4)**						
Dis25: I often talk to others while checking what's on my phone				0.736	0.318	0.545
Dis24: I often walk and use my phone at the same time				0.467	0.268	0.352
Dis21: I can easily follow conversations while using my phone				0.406	0.201	0.310
Dis19: I use several applications on my phone while working				0.334	0.363	0.418

*Percentage of the Total Variance Explained =59.62%. Four factors were extracted from the EFA after 6 iterations*.

Removed items from each subscale due to low loadings:

*F1: Dis5, Dis6*.

*F2: Dis26, Dis29,Dis32, Dis33*.

*F3: Dis8, Dis9, Dis10, Dis11, Dis12, Dis14, Dis15, Dis18*.

*F4: Dis20,Dis22,Dis23*.

*SDS, Smartphone Distraction Scale; ω, McDonald's Omega; α, Cronbach's Alpha; Dis, Items (i.e., Dis1, Dis2); F1, Factor 1; F2, Factor 2; F3, Factor 3; F4, Factor 4*.

The four latent factors comprising of 16 items (Appendix 2) were labeled as*, “Attention Impulsiveness,”* “*Emotion Regulation,” “Online Vigilance,”* and “*Multitasking*.” Furthermore, the first factor (*Attention Impulsiveness*) measures distraction from notifications and smartphone applications as well as the device itself and explained 32.42% of variance. The second factor (*Emotion Regulation*) measures distraction as a coping mechanism for poor mood or distraction as an avoidance mechanism to relieve tension, stress, and anxiety and explained 10.19% of variance. The third factor (*Online Vigilance*) measures distraction due to checking content or preoccupation about checking or if personal online content has been validated, and explained 9.28% of variance. The final factor (*Multitasking*) measures using several smartphone applications while working or walking and using the phone at the same time, and explained 7.72% of variance. Further assessment of the suitability of each item was done by checking the cross-loadings and it was found that the factor loadings were high on their respective constructs.

#### Confirmatory Factor Analysis

**Figure 2:**
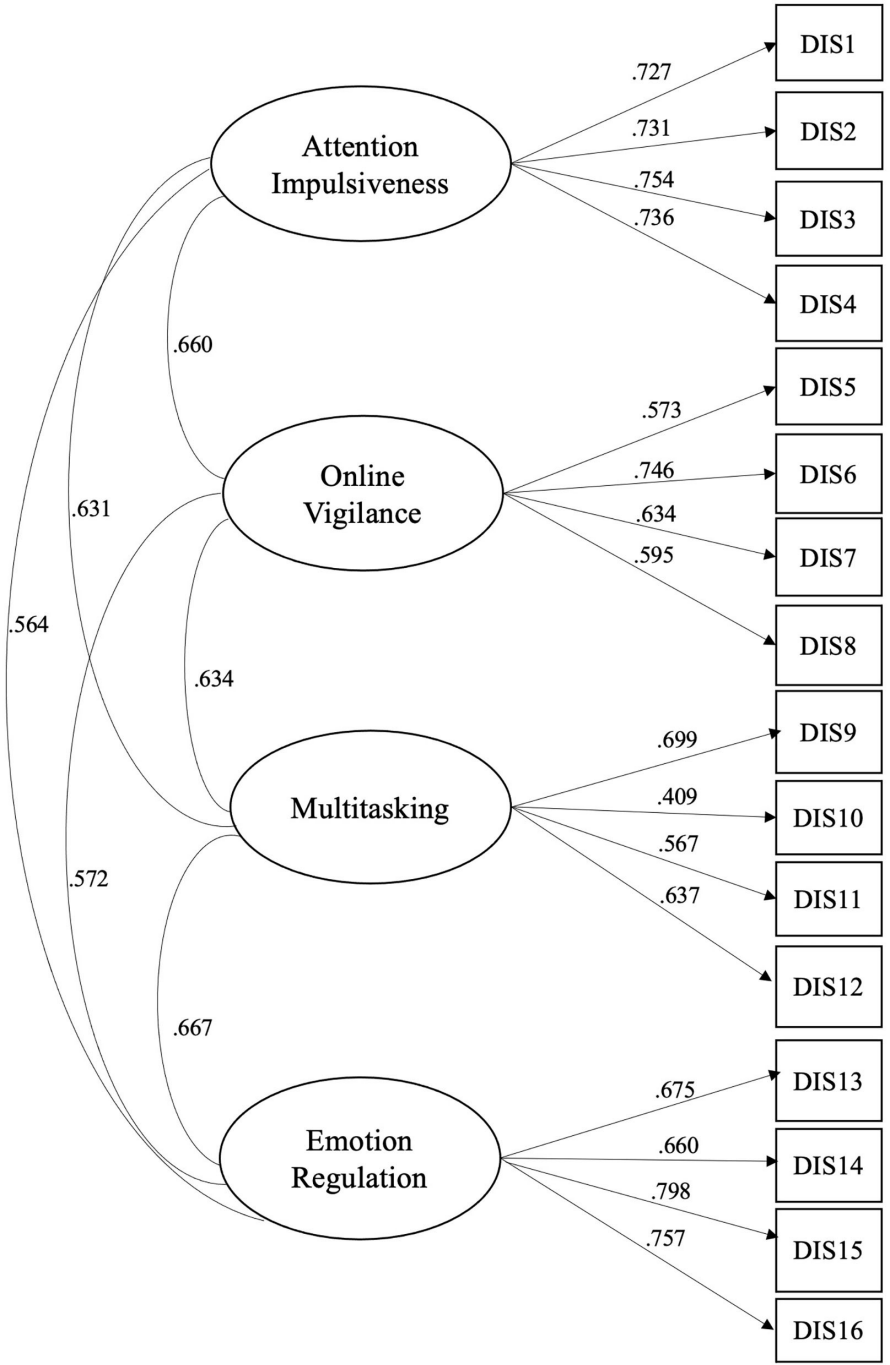
Smartphone distraction scale (SDS) four factor model.

The CFA was used to determine how the data from Sample 2 conformed to the factor structure found in Sample 1. Model fit indices indicated adequate fit for the four-factor model [χ^2^ = 233.56, df = 98; *p* < 0.001; χ^2^/df = 2.38; RMSEA = 0.053; 90% CI (0.044, 0.061), CFI = 0.940; TLI = 0.927, SRMR = 0.044]. All factor loadings of the SDS were statistically significant (*p* < 0.001) and items related to the latent factor (Table 2) (216, 217). Due to high intercorrelations among the four latent factors (Figure 2), an alternative model, a second-order (hierarchical) factor model, was examined to ascertain whether it fitted the data better than the four-factor model. This model examined four latent variables as a function of one general higher-order factor. The results from the analysis showed the following statistics: χ^2^ = 238.28, df = 100, *p* < 0.001; RMSEA = 0.053; 90% CI. (0.044–0.061), CFI = 0.939; TLI = 0.927; SRMR = 0.045. As can be seen, all fit indices suggest that the second-order factor model also fits the data adequately.

**Table 2:** Summary of Confirmatory Factor Analysis results obtained from the 16 items of the Smartphone Distraction Scale (SDS) on Sample 2 (*n* = 500).

**Factors/Items**	**Factor Loadings**
**ATTENTION IMPULSIVENESS**	
I get distracted by my phone notifications.	0.727
I get distracted by my phone apps.	0.731
I get distracted by just having my phone next to me.	0.754
I get distracted by my phone even when my full attention is required on other tasks	0.736
**ONLINE VIGILANCE**	
I get anxious if I don't check messages immediately on my phone	0.573
I think a lot about checking my phone when I can't access it	0.746
I get distracted with what I could post while doing other tasks	0.634
I get distracted thinking how many likes and comments I will get while doing other tasks	0.595
**MULTITASKING**	
I use several applications on my phone while working	0.699
I can easily follow conversations while using my phone	0.409
I often walk and use my phone at the same time	0.567
I often talk to others while checking what's on my phone	0.637
**EMOTION REGULATION**	
Using my phone distracts me from doing unpleasant things	0.675
Using my phone distracts me from negative or unpleasant thoughts	0.660
Using my phone distracts me from tasks that are tedious or difficult	0.798
Using my phone distracts me when I'm under pressure	0.757
**Instructions:** “Below is a collection of statements about your everyday experience with your smartphone. Using the 1–5 scale below, please indicate how often you currently have each experience. Please answer according to what best reflects your everyday experience.” All factor loadings were statistically significant (p < 0.001)	

To decide which of the compared models best approximate the data, we used two well-known criteria: the Akaike Information Criterion (AIC) and the Bayesian Information Criterion (BIC), since the two models were not nested. Typically, the model with the smallest AIC and BIC values is the “best” model. However, if we want to assess the strength of evidence for each candidate model, we could use the ΔAIC and ΔBIC indices (218). ΔAIC and ΔBIC is simply the difference between a model's AIC and BIC (named candidate model and denoted as AICm and BICm) and the model with the smallest AIC and BIC among the compared models (denoted as AIC^*^ and BIC^*^). Both, ΔAIC and ΔBIC can be used as evidence against a candidate model being the best model. According to Fabozzi and colleagues (218) if a ΔAIC and ΔBIC is <2, it is not worth more than a bare mention. In our case, the ΔAIC is 1.657 and ΔBIC is 6.772.

As can be seen, in terms of the AIC index the four-factor model appears to fit the data better than the competing model (second-order). In terms of the BIC index, the ΔBIC value suggests that the difference between the two models is also small (i.e., 6.772), although no clear decision can be made about which model fits the data better. However, based on the principle of parsimony (219), we concluded that the four-factor model fitted the data better than the second-order factor model.

#### Criterion-Related, Convergent, and Divergent Validity

The criterion-related validity of the SDS was assessed by examining participants' test scores on the SDS in relation to daily smartphone use and social media use. As expected, a small positive association between SDS and daily social media use and smartphone use was observed. Convergent validity [the assessment of the level of correlation with a conceptually similar measure (220)] was met with partial correlations with the ACS, MAAS, and MGQ. As shown in Table 3, the SDS showed significant negative moderate correlations with the ACS (*r*[500] = −0.365, *p* < 0.001) and the MAAS (*r*[500] = −0.514, *p* < 0.001). Correlations of the SDS with the BIS-8, DSR, and BSMAS were assessed. The highest correlation was observed with BSMAS (*r*[500] = 0.595, *p* < 0.001), followed by DSR (*r*[500] = 0.470, *p* < 0.001). Moreover, moderate correlations were observed between the SDS and negative metacognitions (r [500] = 0.376, *p* < 0.001) and positive metacognitions (*r*[500] = 0.300, *p* < 0.001) and PSS (*r*[500] = 0.271, *p* < 0.001). Divergent validity was assessed by examining the correlation with the GSE (*r*[500] = 0.002, *p* = 0.675).

**Table 3:** Correlations of the Smartphone Distraction Scale (SDS) With Other Scales: Criterion-related Validity, Convergent, and Discriminant Validity (*n* = 500).

**Scale**	**Correlations**	**Cronbach's α**
Daily recreational social media use	0.171^**^	-
Daily recreational smartphone use	0.148^**^	-
Attentional control	−0.365^**^	0.80
Mindful attention and awareness	−0.514^**^	0.90
Meta-cognition (positive)	0.300^**^	0.90
Meta-cognition (negative)	0.376^**^	0.89
Social media addiction	0.595^**^	0.84
Impulsivity	0.207^**^	0.77
Deficient self-regulation	0.470^**^	0.89
Stress	0.271^**^	0.68
Self-efficacy	0.002	0.86

** *p < 0.001*.

#### Reliability

Cronbach's alpha (α) was calculated for each of the subscales in order to assess internal consistency (162, 163), with a high alpha value indicating that items in the scale assess the same latent factor. Given the multidimensionality of the construct (221) and the limitations of the Cronbach's alpha [see (222)], an alternative internal consistency reliability coefficient was calculated for each subscale, the McDonald's Omega (ω) (223), which according to some scholars provides more accurate reliability findings for applied research (222, 224, 225). Cronbach's alpha coefficients with values of α ≥ 0.70 were considered to reflect adequate reliability with an item-total correlation between 0.25 and 0.75 (226). For McDonald's Omega, threshold values of ω ≥ 0.70 to 0.90 were considered adequate (221). The response form is a 5-point Likert scale ranging from “*almost never*” to “*almost always*,” where high scores represent higher levels of SD. Cronbach's α for the overall SDS (α = 0.87) suggested a high level of internal consistency and therefore these four factors are strong indicators of the construct. The four subscales presented acceptable to good reliability: Cronbach's alpha for Attention Impulsiveness (α = 0.84), was followed by Emotion Regulation (α = 0.80), Multitasking (α = 0.75), and Online Vigilance (α = 0.74). More specifically, for Sample 1 (*n* = 501) the Cronbach's alpha was α = 0.87, whereas for Sample 2 (*n* = 500) was α = 0.86. McDonald's Omega was highest for Online Vigilance (ω = 0.83), followed by Attention Impulsiveness (ω = 0.78), Emotion Regulation (ω = 0.74), and Multitasking (ω = 0.63).

#### Testing for Measurement Invariance Across Gender

##### Configural Invariance

Configural invariance tests whether the same number of factors are prevalent in both genders (i.e., a four-factor model) and whether the same items load to each factor (i.e., same pattern of fixed and free loadings) across groups. Measurement invariance of the model for gender was tested through estimating the SDS model separately for male and female young adults by constraining the basic latent structure to equality across groups (227). The fit indices of the unconstrained models (see Table 4) demonstrated configural invariance across gender (χ^2^ [196] = 340.014, *p* < 0.001, CFI = 0.935, TLI = 0.921, RMSEA = 0.054 [0.044–0.064], SRMR = 0.051) and therefore an adequate fit for both gender groups. This suggested that both genders had the same basic conceptualization of SD and interpreted the items of each factor similarly.

**Table 4:** Fit indices for multi-group confirmatory factor analysis evaluating measurement invariance of the four factor structure of the SDS (*n* = 500).

**Models Males vs. Females**	**χ^2^**	**df**	**CFI**	**TLI**	**RMSEA**	**90% CI**	**SRMR**	**Model**	**ΔCFI**	**ΔRMSEA**	**ΔSRMR**
Configural invariance	340.014*	196	0.935	0.921	0.054	[0.044–0.064]	0.051	-	-	-	-
Metric invariance	347.700*	208	0.937	0.927	0.052	[0.042–0.061]	0.053	2 vs. 1	0.002	0.002	0.002
Scalar invariance	367.237*	220	0.934	0.928	0.052	[0.042–0.061]	0.054	3 vs. 2	0.003	0.000	0.001

Each model compared with the previous model *p < 0.001.

*n, sample size; χ^2^, chi-square; df, degrees of freedom; CFI, Comparative Fit Index; RMSEA, The Root Mean Square error of Approximation; SRMR, Standardized Root Mean Square Residual*.

##### Metric Invariance

Following configural invariance, metric invariance was evaluated to determine if the strength of the factor loadings of the respective items were equivalent in both groups. A lack of metric invariance could signal a different attribution of importance of certain items or that there is a different understanding of certain items amongst the two groups (228). To assess metric invariance factor loadings are further constrained across groups by choosing an item to serve as a referent metric for each factor with subsequent steps to ensure that the referent item itself is invariant across the two samples. To achieve this all other items on the subscale serve as temporary references against the target item (210). Metric invariance is established if the change in model fit from the configurally invariant model to the metric model does not exceed the following statistical cut-offs:, CFI ≥ −0.010 and RMSEA ≥ 0.015, or SRMR ≥ 0.030 (213). Therefore, a model was tested in which the unstandardized relationships between the items and factors of the SDS were constrained to be equal across the two genders. This constraining to equality did not lead to a significant reduction in model fit (ΔCFI = 0.002, ΔRMSEA = 0.002, ΔSRMR = 0.002), thus supporting metric invariance implying equal salience of factors for both male and female students (Table 4) (228).

##### Scalar Invariance

Since metric invariance was supported, the third step of measurement was scalar invariance establishing whether mean responses for corresponding items were similar across groups. Scalar invariance tests the equality of intercept terms and is achieved by constraining item intercepts to equality and assessing whether the item loadings and the item intercepts are equivalent. It is established if the change in model fit from the metric invariant model does not exceed CFI ≥ −0.010 and RMSEA ≥ 0.015 or SRMR ≥ 0.030 (213). Scalar invariance is considered valid when comparing latent factor means across groups (229, 230), confirming that both genders respond to the scale similarly (231). Therefore, unless scalar invariance is supported, no valid cross-group comparisons can be attempted. Scalar invariance is also a prerequisite to assessing mean differences between the groups (230, 232). Therefore, to test for scalar invariance all the item intercepts were constrained across groups and results demonstrated that scalar invariance across gender groups was confirmed (ΔCFI = 0.003, ΔRMSEA = 0.000, ΔSRMR = 0.001) (Table 4).

### Testing for Latent Mean Differences

Since the observed item intercepts and the factor loadings of the items were invariant across genders (211), analysis of potential latent means differences were examined (233). A latent mean analysis was therefore performed for SDS among male and female groups by constraining the latent means of the male group (serving as the reference group) to zero, while the mean of the other group was freely estimated (the decision on which group to constrain is arbitrary with no influence on the final estimated mean values) (234). In the case of the SDS, latent means analysis identified statistically significant gender differences between males and females. Positive values suggest that the comparison group (females) have significantly higher scores than the reference group (males) across all latent factors: Emotion regulation (0.405), Attention Impulsiveness (0.507), Online Vigilance (CR = 0.279), and Multitasking (0.348). These results indicate gender differences underlying both cognitive and emotive dimensions of distraction in smartphone use among males and females.

## Discussion

Attention is a scarce resource and fragmented attention appears to be a frequent outcome of smartphone use related to cognitive interference and interruptions (48, 235, 236). Distraction is one expression of attentional loss associated with smartphone use. The present study explored a newly conceptualized, theory-guided, multidimensional measure of SD based on the need to understand and develop a psychometric assessment framework for SD. To achieve this goal, the perceptual control theory (58) and the control model of engagement for social media and smartphone use (52) among young adults were adopted to explain the tendency for distraction in order to control self-presentation, content and relationships online. The present study had the following aims: (i) identify the latent dimensions of SD and develop a respective pool of items, (ii) evaluate the scale's validity and reliability, (iii) investigate the criterion-related, convergent, and divergent validity with existing measures from the smartphone literature, and (iv) establish gender invariance (at the configural, metric, and scalar levels), and test latent mean differences across males and females. The SDS appeared to be a valid and reliable measure for the assessment of SD with sound psychometric properties and invariance across gender among young adults. Results from the measurement invariance analysis supported the configural, scalar, and metric invariance for the four-factor structure, suggesting that the SDS is comparable across the two groups. Furthermore, latent mean differences indicated that females were more susceptible to SD than males, consistent with the smartphone literature (148, 154, 208).

The analyses conducted provided evidence of the validity of a four-factor structure comprising of attention impulsiveness, emotion regulation, online vigilance, and multitasking and confirming that SD entails a cognitive, emotive, and behavioral component, consistent with the evidence reported in the literature (8, 27, 56, 60, 120, 237, 238). Statistically, the four-factor model was followed with a marginal difference in terms of fit by a hierarchical model, providing further evidence of the multidimensional and multifaceted nature of SD rendering a second-order model (239). However, the more parsimonious solution was chosen as suggested by scholars (240). In the four factor model, as hypothesized, the first factor (*Emotion Regulation)* was the strongest factor referring to strategies individuals use to modulate the emotional state they are in, the timing of the emotion and its expression (241), suggesting that SD has a strong regulating function consistent with literature (242–246). Emotion regulation has been found to be associated with self-control and can be dependent on intrinsic (i.e., temperamental) or extrinsic (i.e., attachment) factors (247) and may be regulated through avoidance, suppression, or enforced expression or reappraisal (241). Within smartphone use, distraction appears to serve a protective function by re-directing attention to a situation of less valence avoiding negative emotional states, consistent with evidence of general distraction and interference in anxiety (248, 249). However, overreliance may be associated with problematic smartphone and social media use (83).

The second factor (*Attention Impulsiveness*) referred to difficulties in the regulation of attention and engagement in impulsive behavior. Impulsivity has been linked to temporal discounting of rewards driven by emotion regulation and presenting as reaction to emotional arousal (250). Distraction frequency has been associated with attention impulsiveness, which is triggered by anxiety and takes the form of attentional bias (23), as has been supported in the smartphone and social media use literature (101, 249, 251). Attention impulsiveness has also been associated with habitual checking (121), chronic media multitasking and attention decrements (12) as well as with impaired disengagement in Internet Gaming Disorder (IGD) (252). In conditions where learning is of low interest, attentional impulsivity is associated with increased interruptions, reduced lecture comprehension, low motivation, and fluid intelligence (35, 36), to the detriment of academic performance and tasks requiring sustained attention (37).

The third Factor (*Online Vigilance*) related to cognitive preoccupation and orientation toward social media content with items reflecting salience (i.e., thinking intensively online spaces), reactivity (i.e., readiness to react to smartphone cues even if it involves interruption of activities), and monitoring (i.e., tendency to actively observe online engagement parallel to other activities) (91). The findings supported a strong relationship between distraction and online preoccupation and vigilance, and may predispose an individual to distract frequently and check digital devices excessively for reassurance (92) and use smartphones more than intended or in a compulsive way (52, 91, 253). Online vigilance therefore, appears potentially fueled by FOMO and associated with disruptions to attend to smartphone content, further corroborating previous findings from the literature reporting regulation deficits in IGD and Problematic Internet Use (PIU) (118, 186, 254, 255). Strong habitual checking behaviors, reinforced by the immediate smartphone access to social media and the disruption of notifications, appear to be leading to self-control failures (125).

The fourth Factor (*Multitasking*) represented general multitasking behavior taking place while using smartphones, which may be associated with a distractive state (237). Task switching requires time investment and mental resources to re-orient to the task at hand with responses being slower and more error-prone (256). Multitasking has been considered as functionally equivalent to distraction (237). However, multitasking may mask the perception of distraction (257). There are reasons to expect a high degree of overlap among the four dimensions, reflected in the high co-variances amongst the factors as well as in the error terms of specific items. All dimensions measured distraction within smartphone use and had an implicit or explicit focus on cognitive preoccupation with smartphone content (primarily social media content, for emotion regulation and resulting attention loss, potentially leading to checking and multitasking), in accordance with evidence (12, 23, 24, 237, 258–260). Therefore, the overlap and the high inter-correlation amongst the factors was expected. However, recent evidence on highly prevalent non-social smartphone and process use (e.g., watching videos, browsing online) (73) has been associated with problematic smartphone use (92, 94) and should therefore be taken into account in future studies by including items related to the diverse content that a smartphone provides access to.

To establish the convergent and discriminant validity of the SDS, the study investigated the association between various cognitive, emotional, and behavioral variables and the SDS factors. Criterion-related and convergent validity was demonstrated through associations with daily smartphone and social media use, attentional control and mindful attention and awareness. Significant correlations were also observed between the four factors of the SDS and corresponding psychological constructs, such as deficits in emotion regulation, problematic social media use, and poor metacognition, thus providing further evidence for the test's convergent validity and bridging research on IGD and PIU with social media and smartphone use in identifying common risk factors and potential outcomes (118, 186, 254, 255, 261, 262). Therefore, the SDS appears to demonstrate acceptable validity and reliability.

Additionally, the present study aimed to assess measurement invariance of the SDS across gender. The findings obtained suggested that the SDS factor structure is the same across gender with equally robust associations between the underlying constructs and the observed indicators across genders, thus providing additional support for the four-factor structure of the SDS. In addition, the SDS achieved both metric and scalar invariance, suggesting equal salience of the indicators across the two groups, providing additional evidence of construct validity for cross-group comparisons for the SDS. As suggested in previous literature, measurement invariance needs to be supported before any cross-cultural investigations of the scale are attempted (231). Although the SDS demonstrated measurement invariance, findings suggested that the latent means for the SDS subscales differed across gender groups. Latent mean differences were assessed by using a latent modeling approach which is considered a more robust approach (when compared to testing mean differences with *t*-tests), providing strong empirical support for gender differences (212). The results from this analysis found that students of both genders were not similar in their endorsement of the SDS subscales, with females exhibiting higher scores than males across all subfactors, contributing to the emerging body of smartphone literature on gender (146, 147, 154).

These results are also in line with findings from previous studies in which females appear to demonstrate higher multitasking and emotion regulation needs, and to manage their emotions more poorly than males and present with higher problematic smartphone use (146, 152, 263–266). Evidence regarding gender differences in multitasking is inconclusive due to conflicting findings, with some evidence suggesting that women are not better than men at multitasking, while other literature suggests that women present with better multitasking skills (151, 267). To explain these differences, the *hunter-gatherer hypothesis* (claiming a cognitive adaptation to different division of labor roles across the sexes) (268) has been proposed to explain findings of females being less affected by task-irrelevant interruptions in experimentally-generated multitasking conditions, suggesting that females are better at multitasking. However, media multitasking is considered the new norm, and inadvertently leads to fragmented attention and frequent micro-disengagements due to interruptions (39), linking multitasking with distraction (269). Still, no direct conclusions may be drawn given the relative absence of research on SD to date. Previous studies examining differences between genders in smartphone use have indicated that females report higher smartphone use and present with greater prevalence of problematic smartphone use (147, 148, 270), which clearly indicates cross-gender differences (271).

To the best of the authors' knowledge, the present study is the first to develop and investigate the psychometric properties of a newly developed measure on SD, as well as to provide evidence regarding measurement invariance across gender. The findings of this study suggest that the SDS functions well and is invariant across genders among young people, providing new insights in the smartphone literature by suggesting cognitive and emotive effects in terms of attentional loss from smartphone use across genders. The SDS presents with a strong theoretical foundation, good psychometric properties, short length, and easy applicability. The findings obtained suggest that the instrument may be used and further tested in the general population when assessing the construct of SD.

The SDS requires further investigation with ethnically diverse samples and different age groups and settings, establishing its test-retest stability, invariance across different cultures, and its predictive validity, by exploring its relationship with other relevant psychological constructs, such as anxiety and mood disorders or attention deficit hyperactivity disorder (ADHD) (272), especially in clinical samples by identifying how the frequency and compulsiveness of smartphone use and the impact of this cognitive-emotive construct may contribute to the deterioration or alleviation of symptoms of various disorders (273). Additionally, the role of SD should be examined in terms of risky behaviors, physical injuries (17, 274), work performance so that greater knowledge about SD may be generated within distinct subgroups and environments. Associations of SD with metacognition for problematic smartphone use should be further explored with the use of validated instruments (116), as no relevant measure was available during data collection of the present study. Therefore, further validation of the construct is required and to encourage research investigating distraction in other contexts.

Potential limitations in the present study include the lack of specific aspects of internal consistency of the scale such as test-retest reliability and its limited generalizability to the broader population, having relied on a convenience self-selected sample of university students, which may not necessarily be representative of all smartphone users. It is unclear how culturally distinct or age different samples (e.g., young children) might respond to this scale. Additionally, the content of the items may warrant further refinement (i.e., the driving item was not relevant among emergent adults). However, SD has been suggested as a common behavior of concurrent smartphone use among older adults (17). Another important potential limitation constitutes the use of self-report questionnaires and potential biases associated with self-report methods (e.g., social desirability, memory recall). Combined with behavioral and biometric data, psychometric measures of SD as both an adaptive but also as a maladaptive digital experience could provide strong evidence of face validity. Additionally, the construct of SD does not encompass other experiences of distraction on other digital devices or media multitasking or process smartphone use. Smartphones were chosen because they are the most ubiquitous and pervasive devices. Such insight would make it possible to discern whether the nature of distraction similarly to online addiction varies between platforms, digital devices, and content types (275). Future studies may consider including items related to media multitasking and overall digital distraction arising from using multiple devices may provide a more inclusive account of the digital experience. The present study and its findings support the use of the SDS four factor model. However, the present study did not test equivalence for the hierarchical model. Still, the adequate fit of the hierarchical model, which was marginally inferior to the first order, suggests a strong general factor representing the construct of smartphone distraction. Thus, when calculating scores, authors are advised to work with subscale scores or use a total score. However, given that the the hierarchical model was not tested for invariance in this preliminary investigation, which focused primarily on the development and initial validation of the scale, invariance testing of the second order model and latent mean differences is strongly recommended to be tested in a future study to support equivalence across genders and assess gender differences in the hierarchical model. The first step of invariance in the four factor model, which is a prerequisite to testing invariance of the hierarchical model has been satisfied in the present investigation.

The findings obtained suggest that the SDS is a psychometrically sound scale assessing SD guided by two theoretical frameworks according to which cognitive preoccupation and need to control content, relationships, and self-presentation appear to be key drivers for distraction via smartphone use. The SDS was designed to be applicable to young adult smartphone users irrespective of level of smartphone use, whether excessive or judicious. The SDS may be utilized as a screening tool in interventions to reduce the risk of problematic smartphone use in student populations (276). Given that smartphones are ubiquitous, SD is a common behavior, impacting productivity and areas of executive function (277), and therefore reducing distraction may be of particular importance to aid and enhance performance, emotion regulation, and overall psychological well-being.

## Conclusion

Attention management may be one of the most critical skills of this century where information is abundant. Attention is a scarce resource and its control may be impaired by the online environment and digital devices available. Distraction is invariably part of an individuals' online and offline experiences. The present study sought to devise the first SDS and further investigate its psychometric properties, given the absence of a similar construct in the smartphone literature. The SDS is best conceptualized within a four-factor solution. Additionally, the SDS was found to present with gender measurement invariance at the configural, metric, and scalar levels, suggesting that the scale functions equivalently across the two gender groups. Moreover, latent mean analysis indicated gender differences underlying both cognitive and emotive dimensions of distraction in smartphone use. The SDS is a theory-guided scale, with sound psychometric properties assessing a complex psychosocial construct defined by cognitive-emotive dimensions with positive and negative valence related to attention impulsiveness, emotion regulation, online vigilance, and multitasking. Within the smartphone literature, SD is an emergent issue interfering with everyday functioning and productivity and potentially implicated in problematic smartphone and social media use.

## Data Availability Statement

The raw data supporting the conclusions of this article will be made available by the authors, without undue reservation.

## Ethics Statement

The studies involving human participants were reviewed and approved by Nottingham Trent University College of Business, Law and Social Sciences. The patients/participants provided their written informed consent to participate in this study.

## Author Contributions

MT: principal investigator, main author, study design, data collection, and statistical processing of the data. HP and IT: statistical and methodological supervision. MG, MR, DK, and HP: supporting the study design and supervision of the study. MT, HP, IT, MG, MR, and DK: editing the manuscript. All authors contributed to the article and approved the submitted version.

## Conflict of Interest

The authors declare that the research was conducted in the absence of any commercial or financial relationships that could be construed as a potential conflict of interest.
