# Supplementary figures and images for: Exploring the Dimensions of Smartphone Distraction: Development, Validation, Measurement Invariance, and Latent Mean Differences of the Smartphone Distraction Scale (SDS)
Source: Front Psychiatry. 2021 Mar 8;12:642634. doi: 10.3389/fpsyt.2021.642634 (PMC7982468; doi:10.3389/fpsyt.2021.642634)

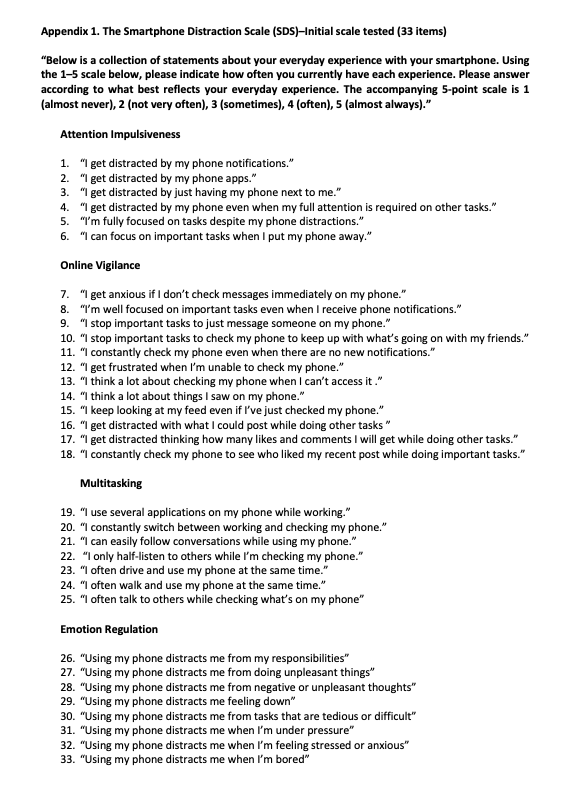

Supplement: Supplementary file 1 [file Image_1.png]

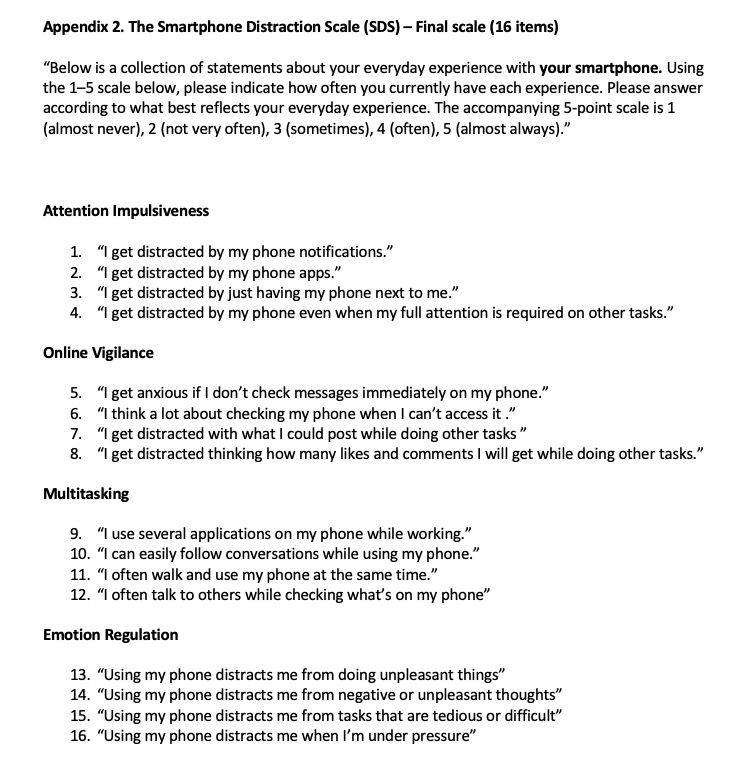

Supplement: Supplementary file 2 [file Image_2.png]
